# Locomotor Ataxy

**Published:** 1889-11-02

**Authors:** 


### LOCOMOTOR ATAXY.

A somewhat lengthy article is contributed to the *Therapeutic Gazette*, Philadelphia, for September, by Prof. Dujardin-Beaumetz, on "Suspension in Locomotor Ataxy.' "Many years ago extension was recommended for the cure of tabes. . . . But the operations of stretching the sciatica nerve for tabes has been abandoned." Yet the operation comes back under another form—viz., that of suspension. Out of twenty-five patients good results have been obtained in most cases, and in four a real improvement in the walking. In proof of this diagrams are given showing the walking power of the ataxic before and after suspension. The author has never seen any harm result from the treatment, but it is not to be forgotten that Goreki reported a case of death from suspension, but the person caused himself to be suspended by his own servant, and no medical man was present. The *modus operandi* is simple, and woodcuts are appended, showing how the suspension apparatus is applied. A strap under the chin and under the occiput connected at the side of the head is used, and the patient is first suspended for thirty seconds, then for three minutes. It is not absolutely necessary to lift the patient from the floor; it is sufficient that the tips of the toes shall touch the floor. The face becomes congested, sometimes cyanosed, "and you ought always to be ready to lessen the traction if the patient begins to be distressed." The apparatus may also be supplemented by straps for the armpits. A woodcut is given, showing various kinds of apparatus, and the movements which may be executed under their use. And the author mentions the modification devised by Dr. Weir Mitchell. Dr. Mitchell's apparatus consists of two leathern slings for the elbows, while a band confines the arms to the waist. The patient is suspended, partly by the head and partly by the elbows. To distribute the pressure on the arms and trunk, the vertical straps are widened under each elbow to about four or five inches. Dr. Mitchell affirms that by his apparatus suspension may be maintained for more than twenty minutes. In reply to the query, how does suspension act? the author remarks that it acts as a process of stretching, which brings about a modification in the nutrition of the cord, and in particular in those of its posterior columns. The contra-indications of the treatment are arterio-sclerosis and organic heart disease.

In the *British Medical Journal* for October 19, Dr. Julius Althaus, writing of suspension for locomotor ataxy, oberves "Everything depends on the method of doing it," and he comments on a case recorded by Dr. Churton, where suspension was at first extended to five minutes. Dr. Althaus soon found, when beginning to practice this treatment, that the best results are obtained with short suspensions frequently repeated. He also considers it bad to suspend by the occiput and chin, and takes care that the patient is well supported by the armpits also.

We also perceive some notes given by Dr. J. S. Risien Russell and Dr. James Taylor of "cases treated by suspension" at the National Hospital for Paralysed and Epileptic, at Queen-square.* The cases recorded are all tabes dorsali, with the exception of two, one of lateral sclerosis, and the other of postero-lateral sclerosis, or the "ataxic paraplegia" of Dr. Gower. Suspension was used every other day. Half a minute to a minute was the time allowed for the first suspension, and the time was afterwards increased up to four minutes. Both arm and head pieces were used, and as soon as the patient had acquired confidence he was directed to raise the arms slowly from the sides until the whole weight of the trunk was thrown on the neck. The inconveniences experienced by the patients were in most cases slight. Pain at the back of the neck, soreness of the jaws, and pain at the bottom of the back were the principal complaints. But one or two patients also complained of faintness during the suspension. As to the result of the treatment, all that the reporters are justified in saying is that their patients improved. One improved at first, but when he left the hospital was certainly worse. Another case, after twenty-five suspensions, experienced no improvement. A month afterwards the ataxy was worse. After ten more suspensions he began to improve, and then left the hospital. The authors observe that "considering the negative results of the first course of suspensions, the improvement during the second course can scarcely be regarded as anything more than a coincidence." Where improvement did occur, it consisted in a less ataxic gait and greater steadiness in standing. The case of lateral sclerosis became worse. The case of postero-lateral sclerosis only received four suspensions, as epigastric pain and nausea coming on during each suspension it was not considered desirable to persist in the treatment. Altogether sixteen cases are recorded, and the record is certainly not sufficiently encouraging to tend to establish this manner of dealing with ataxy.

* *Lancet*, Oct. 19, 1889.

